# Effects of Endurance Training on the Coenzyme Q Redox State in Rat Heart, Liver, and Brain at the Tissue and Mitochondrial Levels: Implications for Reactive Oxygen Species Formation and Respiratory Chain Remodeling

**DOI:** 10.3390/ijms23020896

**Published:** 2022-01-14

**Authors:** Karolina Dominiak, Lukasz Galganski, Adrianna Budzinska, Andrzej Woyda-Ploszczyca, Jerzy A. Zoladz, Wieslawa Jarmuszkiewicz

**Affiliations:** 1 Laboratory of Mitochondrial Biochemistry, Department of Bioenergetics, Faculty of Biology, Adam Mickiewicz University, 61-614 Poznan, Poland; karolina.ogrodna@amu.edu.pl (K.D.); adrianna.budzinska@amu.edu.pl (A.B.); andrzej.woyda-ploszczyca@amu.edu.pl (A.W.-P.); 2 Chair of Exercise Physiology and Muscle Bioenergetics, Faculty of Health Sciences, Jagiellonian University Medical College, 31-066 Krakow, Poland; j.zoladz@uj.edu.pl

**Keywords:** coenzyme Q, reactive oxygen species, mitochondrial energetics, endurance training

## Abstract

Sixteen adult, 4-month-old male Wistar rats were randomly assigned to the training group (*n* = 8) or the control group (*n* = 8). We elucidated the effects of 8 weeks of endurance training on coenzyme Q (Q) content and the formation of reactive oxygen species (ROS) at the tissue level and in isolated mitochondria of the rat heart, liver and brain. We demonstrated that endurance training enhanced mitochondrial biogenesis in all tested organs, while a significant increase in the Q redox state was observed in the heart and brain, indicating an elevated level of QH_2_ as an antioxidant. Moreover, endurance training increased the mQH_2_ antioxidant pool in the mitochondria of the heart and liver, but not in the brain. At the tissue and isolated mitochondria level, an increase in ROS formation was only observed in the heart. ROS formation observed in the mitochondria of individual rat tissues after training may be associated with changes in the activity/amount of individual components of the oxidative phosphorylation system and its molecular organization, as well as with the size of the oxidized pool of mitochondrial Q acting as an electron carrier in the respiratory chain. Our results indicate that tissue-dependent changes induced by endurance training in the cellular and mitochondrial QH_2_ pool acting as an antioxidant and in the mitochondrial Q pool serving the respiratory chain may serve important roles in energy metabolism, redox homeostasis and the level of oxidative stress.

## 1. Introduction

Physical exercise provides a variety of metabolic, thermal and mechanical stimuli to various organs of the body, leading to a variety of adaptive responses [1]. Acute physical exercise has been shown to increase oxidative stress in skeletal muscles and other organs [2,3,4,5], which can be harmful to the body [2], but in small doses, can stimulate several adaptive responses in different organs [6,7,8]. It is well documented that endurance training has a strong effect on increasing the activity/amount of skeletal muscle mitochondrial enzymes [9,10,11], resulting in the improved metabolic stability of muscles during exercise [12,13] and resistance to fatigue [14]. Much less is known about the effects of endurance training on mitochondrial enzyme activity and the state of oxidants in other vital organs of the body, such as the brain, heart and liver. Acute exercise has been shown to increase the production of oxidants in the aged heart but not in the liver, thereby indicating that aging heart muscle is more susceptible to oxidative stress after heavy exercise than the liver [15]. Moreover, the adaptive responses of the brain to oxidative stress induced by acute or chronic exercise differ significantly from those of the liver and heart as well as fast- and slow-twitch muscles [4]. Nevertheless, there are few studies involving the same animals that show the multiorgan response of mitochondria to endurance training—especially studies focused on the role of coenzyme Q (Q).

Mitochondria are organelles that are crucial for cellular energy production and the formation of reactive oxygen species (ROS) [16]. The reduced form of Q (QH_2_) is an important antioxidant found in all cell membranes, including the mitochondria [17,18,19]. Additionally, the redox state of Q may be a useful marker of cellular oxidative stress. Mitochondrial Q (mQ) is a key nonprotein carrier of electrons in the respiratory chain that is also involved in the production of mitochondrial ROS (mROS), which arises as a byproduct of oxygen metabolism or under oxidative stress conditions [20,21], including acute or chronic/endurance training. However, little is known about the role of mQ in mitochondrial adaptation to endurance training.

The purpose of this study was to investigate the effects of 8-week endurance training on mitochondrial biogenesis, Q content and the formation of ROS in highly energy-dependent rat tissues such as the heart, liver and brain. Research on ROS production and changes in the amounts of the reduced and oxidized Q (Q9 and Q10) pools was performed at the tissue (homogenate) and isolated mitochondria levels. Additionally, we examined the changes induced by endurance training at the level of the components of the oxidative phosphorylation (OXPHOS) system and antioxidant enzymes in isolated mitochondria. Our results show that the endurance training-induced changes in the mitochondria of all examined tissues may serve a role in their energy metabolism, redox homeostasis and level of oxidative stress. Our research highlights the role of a reduced Q pool at both the cellular and mitochondrial levels in protecting against ROS overproduction. It also highlights the role of the oxidized (reducible via the respiratory chain) mitochondrial Q (mQ) pool in adapting the OXPHOS system and modulating mROS production in response to endurance training

## 2. Results

### 2.1. Endurance Training Increases Mitochondrial Biogenesis in the Heart, Liver and Brain

**Figure 1:**
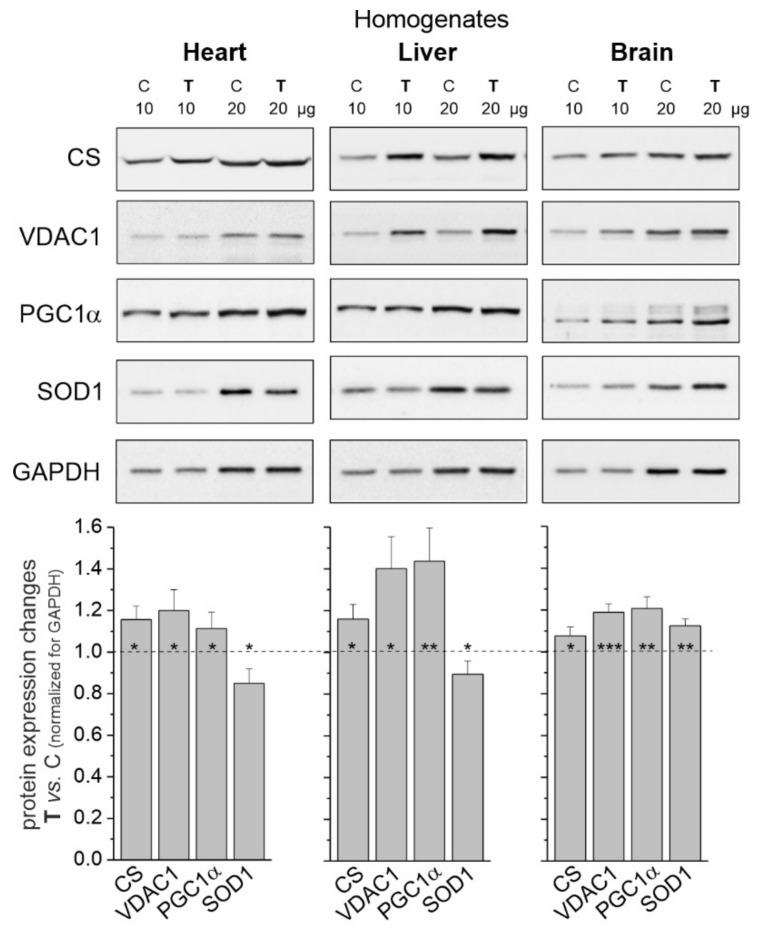
Representative Western blots and analyses of protein expression in rat heart, liver and brain homogenates from control (C) and trained (**T**) rats. CS, citrate synthase; VDAC 1, voltage-dependent anion-selective channel protein 1; PGC1α, peroxisome proliferator-activated receptor gamma coactivator 1-alpha; SOD1, superoxide dismutase 1; GAPDH, glyceraldehyde 3-phosphate dehydrogenase. Mean ± SD; *n* = 3–4 homogenate preparations (equal to the number of animals used in each group). *p* < 0.05 (*), *p* < 0.01 (**), *p* < 0.001 (***), comparison vs. control values for a given tissue.

Elevated levels of citrate synthase (CS) and voltage-dependent anion channel 1 (VDAC1) (Figure 1) indicate increased mitochondrial biogenesis in all tissues tested in trained rats. As a result of an 8-week endurance training, the greatest increase in mitochondrial protein levels was observed in the liver (~30% mean increase), while this increase was smaller in the heart and brain (~17 and ~12%, respectively). Additionally, endurance training induced significantly higher expression levels of peroxisome proliferator-activated receptor γ coactivator 1α (PGC1α), a transcriptional coactivator that regulates the genes involved in mitochondrial biogenesis.

In tissues with a greater increase in training-induced mitochondrial biogenesis (i.e., the heart and liver), a decrease in the level of superoxide dismutase 1 (SOD1) was observed. In contrast, the level of this antioxidant protein increased in the brain (Figure 1).

### 2.2. Endurance Training Increases the Reduced and Oxidized Pools of Q9 and Q10 in the Heart and Liver and Brain

**Figure 2:**
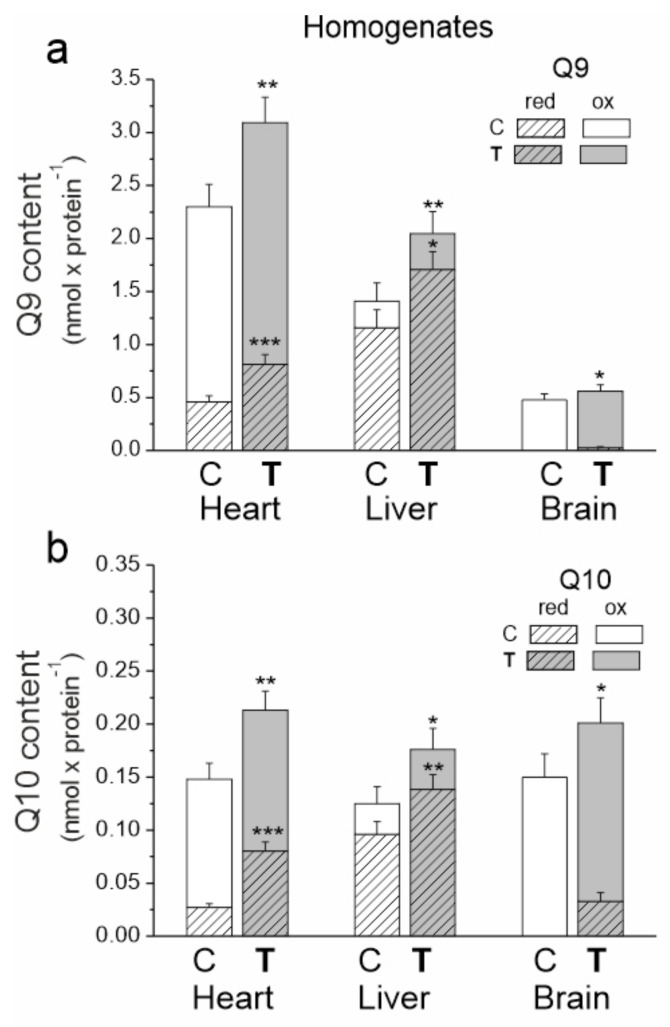
The content of coenzyme Q9 (Q9) (**a**) and coenzyme Q10 (Q10) (**b**) in the heart, liver and brain from control (C) and trained (**T**) rats. The total (Qred + Qox), reduced (Qred) and oxidized (Qox) Q pools were measured under fully oxidizing conditions (no respiratory Q-reducing substrates). Mean ± SD; *n* = 5–6 homogenate preparations (equal to the number of animals used in each group). *p* < 0.05 (*), *p* < 0.01 (**), *p* < 0.001 (***), comparison vs. control values of Qred or Qtot for a given tissue.

In the tested tissues of both trained and untrained rats, the size of the total Q10 pool (reduced plus oxidized) appeared to be similar (Figure 2). However, there were significant differences in the size of the total pool of the dominant Q form (Q9), which was largest for the heart and the smallest for the brain. The sizes of the reduced pools of Q9 and Q10 were greatest in the liver and smallest in the brain, indicating a high need for reduced Q as an antioxidant in the liver and low need in the brain.

In all tested tissues, endurance training increased the total, reduced and oxidized pools of both Q forms (Figure 2). The total Q9 pool increased by ~35, ~45 and 17% in the heart, liver and brain, respectively. Moreover, the total Q10 pool increased by ~44, ~41 and ~34% in the heart, liver and brain, respectively. Reduced Q pools increased by ~77 (Q9) and ~190% (Q10) in the heart and by ~47 (Q9) and ~44% (Q10) in the liver. A significant increase in the reduced Q9 and Q10 pools was also observed in the brain. Since the reduced pools of both Qs were not detected in the brain before training, the percent increase could not be calculated. Given the difference in the size of the Q9 and Q10 pools, the amount of reduced Q9 increased the most in all examined tissues. Additionally, increases in the oxidized Q9 (~24, ~35 and ~11%) and Q10 (~12, ~30 and ~12%) pools in the heart, liver and brain, respectively, were observed after training.

### 2.3. Endurance Training Only Increases H_2_O_2_ Formation in the Heart (Tissue Homogenate) 

**Figure 3:**
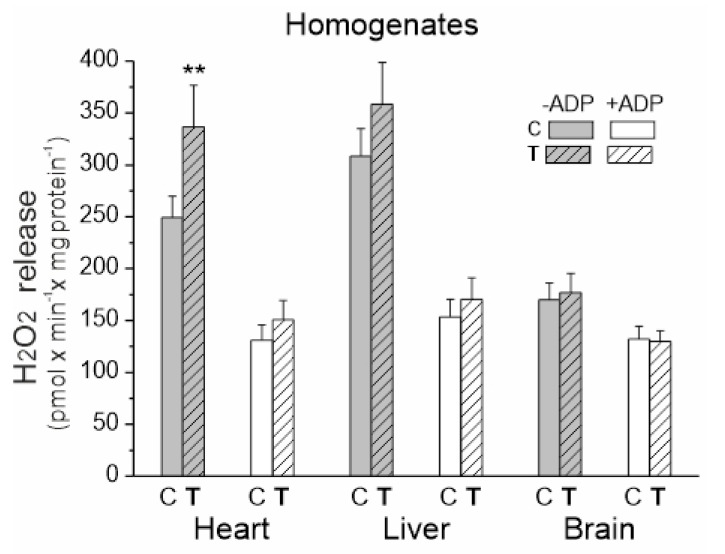
H_2_O_2_ release in heart, liver and brain homogenates from control (C) and trained (**T**) rats. Measurements were performed in the absence or presence of ADP with succinate and malate plus glutamate as mitochondrial respiratory substrates. Mean ± SD; *n* = 5–6 homogenate preparations (equal to the number of animals used in each group). *p* < 0.01 (**), comparison vs. control values for a given tissue.

We measured the rate of H_2_O_2_ release in tissue homogenates under mitochondrial respiratory chain activation in the presence of succinate and malate plus glutamate as respiratory substrates for complex II (CII) and complex I (CI), respectively (Figure 3). Measurements were made in the presence or absence of ADP (i.e., activation or inactivation of the mitochondrial OXPHOS).

Upon comparing the three examined tissues, the level of H_2_O_2_ production under OXPHOS activation conditions was similar for both trained and untrained rats (Figure 3). In the absence of ADP, the release of H_2_O_2_ was significantly higher when compared to the OXPHOS activation conditions, with the highest levels observed in the heart and liver homogenates from both trained and untrained rats. A statistically significant increase in H_2_O_2_ production was only observed in the heart homogenates under inactive OXPHOS conditions.

Further studies were conducted on the level of mitochondria isolated from the heart, liver and brain of trained and untrained rats.

### 2.4. Endurance Training (i) Increases the Reduced and Oxidized mQ Pools in the Heart Mitochondria, (ii) Increases the Total Reduced mQ Pool (QH_2_9 + QH_2_10) and Decreases the Total Oxidized mQ Pool (Q9 + Q10) in the Liver Mitochondria and (iii) Does Not Alter mQ Pools in the Brain Mitochondria 

**Figure 4:**
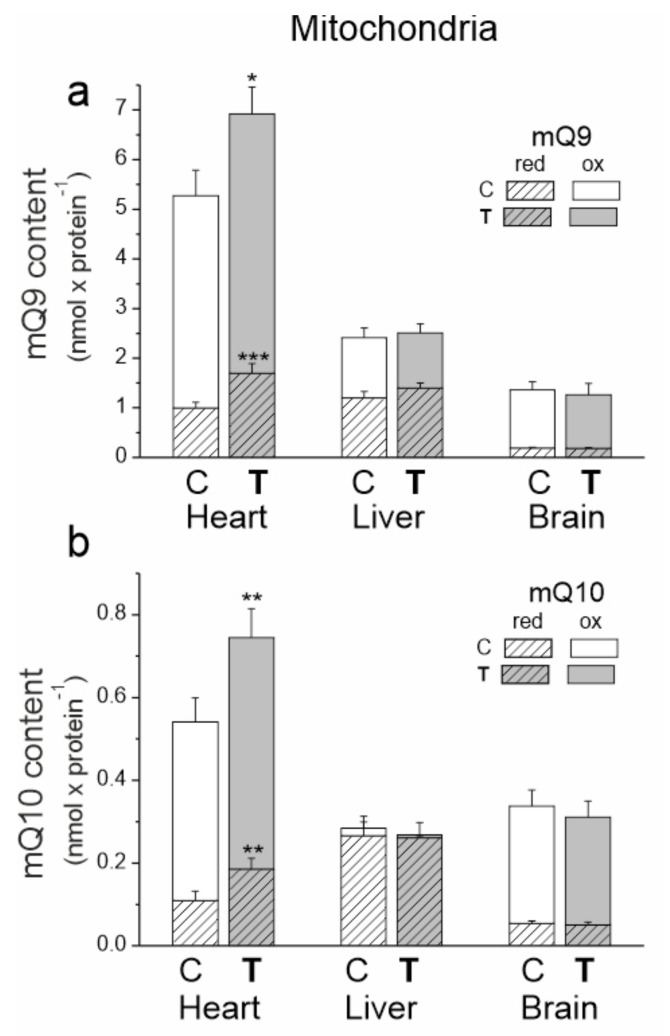
The content of coenzyme Q9 (Q9) (**a**) and coenzyme Q10 (Q10) (**b**) in the heart, liver and brain mitochondria from control (C) and trained (T) rats. The total (Qred + Qox), reduced (Qred) and oxidized (Qox) Q pools were measured under fully oxidizing conditions (no respiratory Q-reducing substrates). Mean ± SD; *n* = 5–6 mitochondrial isolations (equal to the number of animals used in each group). *p* < 0.05 (*), *p* < 0.01 (**), *p* < 0.001 (***), comparison vs. values of Qred or Qtot of control mitochondria for a given type of tissue.

The heart mitochondria of untrained and trained rats had significantly greater amounts of mQ9 and mQ10 than the mitochondria of the other tested tissues (Figure 4). The liver mitochondria had the highest percentage of reduced mQ pools (~50% of the mQ9 pool and ~90% of the mQ10 pool).

The greatest response to endurance training at the mQ pool levels was observed in the mitochondria of the heart. After 8 weeks of training, significantly higher amounts of reduced mQ (mQH_2_9 by ~66% and mQH_2_10 by ~70%) and smaller increases in oxidized mQ (mQ9 by ~15% and mQ10 by ~30%) were observed in the heart mitochondria (Figure 4). Thus, while these changes indicate an increase in the oxidized Q pool required for respiratory chain function, they also indicate a much greater increase in the reduced Q pool that can serve as an antioxidant.

In the mitochondria of the liver, the importance of training-induced changes was evident when mQ9 and mQ10 pools were analyzed together. In the liver mitochondria of trained rats, the total reduced mQ pool (mQH_2_9 + mQH_2_10) increased by ~14%, while the total oxidized Q pool (mQ9 + mQ10) decreased slightly by ~10%. These changes indicate a decrease in the mQ pool available for the respiratory chain and an increase in the mQH2 pool acting as an antioxidant. No statistically significant changes were observed in the oxidized and reduced mQ pools in the brain mitochondria of trained animals.

**Figure 5:**
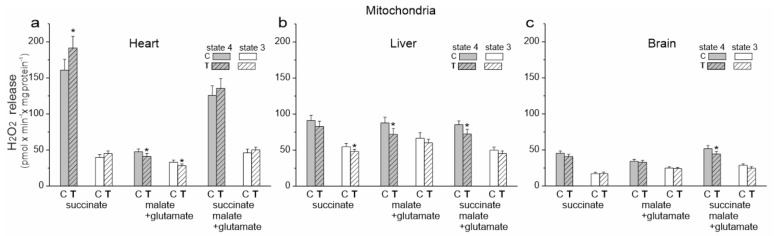
H_2_O_2_ formation release in heart, liver and brain mitochondria from control (C) and trained (T) rats. Measurements were performed with succinate (**a**), malate plus glutamate (**b**) and a mixture of succinate and malate plus glutamate (**c**) under nonphosphorylating (state 4) and phosphorylating (state 3) conditions. Mean ± SD; *n* = 5–6 mitochondrial isolations (equal to the number of animals used in each group). *p* < 0.05 (*), comparison vs. control mitochondria for a given tissue.

Additionally, we investigated changes in the expression level of the mitochondrial Q-binding protein (CoQ10A) required for mQ function in the respiratory chain. In the heart mitochondria, where the pool of oxidized mQ increased after training, the amount of CoQ10A protein decreased significantly. In the mitochondria of the liver, where the pool of oxidized mQ decreased, the level of this protein increased (Figure 5a). In the brain mitochondria of trained rats, the lack of changes in the level of this protein was accompanied by a lack of changes in the pool of oxidized mQ.

### 2.5. Endurance Training Does Not Increase mROS Production in the Liver and Brain Mitochondria but Changes mROS Production Depending on the Respiratory Substrate in Heart Mitochondria

We then examined the release of H_2_O_2_ by the isolated mitochondria of untrained and trained rats during the oxidation of succinate (CII substrate), malate plus glutamate (CI substrates) and a mixture of all of these substrates under phosphorylating (state 3) and nonphosphorylating (state 4) conditions (Figure 5). The involvement of specific sites in the mitochondrial respiratory chain in mROS production is highly dependent on oxidized substrates [22]. Therefore, for a given type of mitochondria and a given respiratory state, a different production of H_2_O_2_ during the oxidation of different respiratory substrates was observed (Figure 5). Under our experimental conditions, during succinate oxidation, the flavin site of CII (site II_F_) and the Q_o_ and Q_i_ sites of CIII (site III_Qo_ and site III_Qi_) could participate in mROS production. Since no rotenone was used, reverse electron transfer from CII to Cl could occur during the oxidation of the succinate. When the malate (plus glutamate) was oxidized, all mROS production sites mentioned could be active, as well as the flavin-dependent site (I_F_) and the mQ-binding site (I_Q_) of CI. However, the purpose of our research was not to determine the contribution of individual mROS production sites but to determine the total mROS production when electrons enter the respiratory chain via CI and/or CII under phosphorylating and nonphosphorylating conditions for the mitochondria of various tissues from control and trained rats.

**Figure 6:**
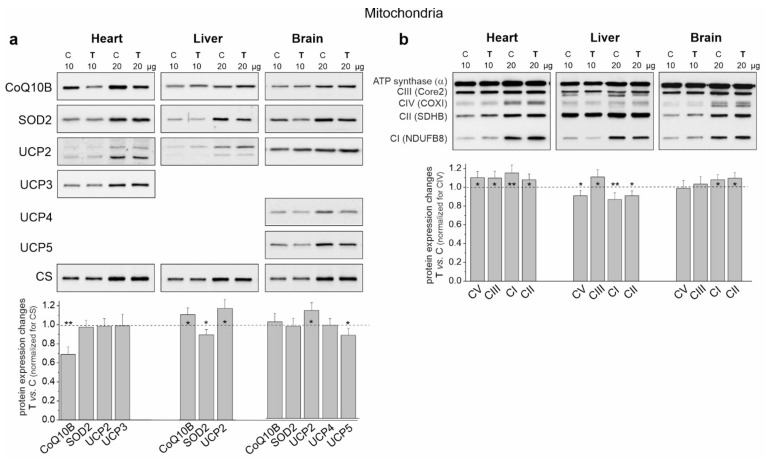
Representative Western blots and analyses of protein expression in the heart, liver and brain mitochondria from control (C) and trained (T) rats. (**a**) CoQ10B, mitochondrial coenzyme Q-binding protein CoQ10 homolog B; SOD2, superoxide dismutase 2; UCP2-5, uncoupling protein 2–5; CS, citrate synthase. (**b**) OXPHOS complexes: CI-CIV, complexes of the respiratory chain; CV and ATP synthase. Mean ± SD; *n* = 3–4 mitochondrial isolations (equal to the number of animals used in each group). *p* < 0.05 (*), *p* < 0.01 (**), comparison vs. control mitochondria for a given tissue.

In the case of cardiac mitochondria among the tested substrates, the highest release of H_2_O_2_ was observed during succinate oxidation under nonphosphorylating conditions for both groups of rats (Figure 5a). Under these conditions, endurance training induced a statistically significant increase in H_2_O_2_ release. During the oxidation of CI substrates, a statistically significant reduction in H_2_O_2_ release in both energy states was observed in the cardiac mitochondria of trained rats. However, a slight non-statistically significant increase in H_2_O_2_ release was observed during the oxidation of the substrate mixture. These results indicate that mROS production could increase in the heart mitochondria of trained rats depending on the involvement of individual respiratory chain dehydrogenases. However, levels of mitochondrial antioxidant proteins such as superoxide dismutase 2 (SOD2) and uncoupling proteins (UCP2 and UCP3) remained unchanged (Figure 6a). 

An overall decrease in H_2_O_2_ release was observed in the liver mitochondria of the trained rats and it was statistically significant under phosphorylating conditions during succinate oxidation and under nonphosphorylating conditions during the oxidation of malate plus glutamate and the mixture of CI and CII substrates (Figure 5b). Additionally, although decreased SOD2 levels were observed in the liver mitochondria of trained rats, UCP2 levels increased (Figure 6a).

In the brain mitochondria of trained rats, no statistically significant changes in H_2_O_2_ release were observed during the oxidation of CI and CII substrates, which were administered separately (Figure 5c). However, during the oxidation of the mixture of these substrates, the level of H_2_O_2_ release was lower under nonphosphorylating conditions. Moreover, unchanged SOD2 levels and increased UCP2 levels were observed in the brain mitochondria of trained rats (Figure 6a).

To understand the changes in mQ pools and mROS production observed in the mitochondria of the heart, liver and brain of trained rats, we investigated at the changes induced by endurance training in the activity and quantity of individual components of the OXPHOS system and its molecular organization.

### 2.6. Endurance Training Leads to an Alteration in the Molecular Organization of the OXPHOS System in the Heart, Liver and Brain Mitochondria

**Figure 7:**
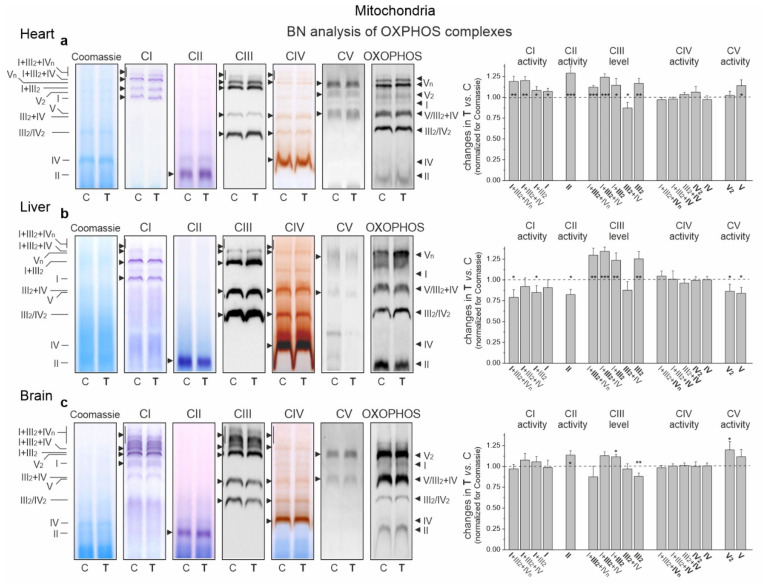
Representative BN-PAGE showing OXPHOS complexes and supercomplexes in mitochondria from the heart (**a**), liver (**b**) and brain (**c**) of control (C) and trained (T) rats. Shown in sequence are Coomassie staining/destaining (loading control), CI in-gel activity, CII in-gel activity, CIII immunoblotting, CIV in-gel activity, CV in-gel activity and total OXPHOS immunoblotting. OXPHOS, oxidative phosphorylation system; CI–CIV, respiratory chain complexes I–IV; CV, ATP synthase. Mean ± SD; *n* = 3–4 mitochondrial isolations (equal to the number of animals used in each group). *p* < 0.05 (*), *p* < 0.01 (**), *p* < 0.001 (***), comparison vs. control values for a given tissue.

In the cardiac mitochondria of trained rats, Western blot analysis showed a statistically significant increase in the expression level of all OXPHOS complexes except CIV (Figure 6b). Additionally, BN-PAGE followed by in-gel activity assays revealed that after endurance training, the activities of CII, CV (V) and all supercomplexes of CI increased (Figure 7a). Moreover, a Western blot of CIII supercomplexes separated by BN-PAGE showed increases in their levels except for III_2_ + IV. All of these changes—along with the increase in the pool of oxidized mQ described earlier (Figure 4a)—indicate a general upregulation of the OXPHOS system in the heart mitochondria of trained rats. A greater increase in the activity of the I + III_2_ + IV(n) compared to I + III_2_ and I with a decrease in the level of III_2_ + IV (Figure 7a) and an increase in the mQ oxidized pool (~23%) (Figure 4a) may lead to a decrease in mROS production during the oxidation of CI substrates (malate plus glutamate) (Figure 5a).

In the liver mitochondria of trained rats, an overall reduction in the protein level (Figure 6b) and activity (Figure 7b) of dehydrogenases (CI and CII) and ATP synthase (CV) was observed. Western blot of CIII separated by the SDS-PAGE (Figure 6b) and BN-PAGE (supercomplexes, Figure 7b) showed an elevation in its level, including all of the supercomplexes except for unchanged III_2_ + IV. All of these changes, along with an increase in the pool of reduced mQs (Figure 4a), may lead to an overall decrease in mROS formation in the liver mitochondria of trained rats.

Although an increase in CI expression was observed (Figure 6b) in the brain mitochondria of the trained rats, the in-gel activity assay showed no statistically significant increase in the activity of individual supercomplexes (Figure 7c). Endurance training increased the level of protein and activity in CII (Figure 6b and Figure 7c, respectively). Although the overall level of CIII did not change, increased levels of its supercomplexes containing CI were observed (I + III_2_ + IV and I + III_2_), possibly at the expense of III_2_ and III_2_ + IV. While the expression of total CV remained unchanged (Figure 6b), the activity of V2 and V increased slightly (Figure 7c).

## 3. Discussion

One might expect that the amount of ROS produced during physical exercise should be higher in organs with a higher metabolic rate (oxygen consumption per gram of tissue × min^−1^). It is well documented that the metabolic rates of the skeletal muscles, heart, brain and liver vary during exercise. Namely, the consumption of oxygen by human skeletal muscles during exercise can be over 80 times higher than at rest [23]. Moreover, it has been shown that oxygen consumption during exercise, when compared to resting level, increases by ~5–6 times in the heart [24], only ~2 times in the liver [25] and not more than ~0.5 times in the brain [26]. These results suggest that heart tissue should be much more exposed to exercise-induced oxidative stress than the brain and liver tissues. Moreover, as proposed by Liu et al. [4], it seems likely that susceptibility to oxidants, activation of antioxidant enzymes, antioxidant levels and other repair systems may vary from organ to organ. To date, no studies have been performed on the same animals to describe the multiorgan response of mitochondria to endurance training in terms of the role of mQ. Our research showed that 8 weeks of relatively intense endurance training resulted in many changes important to energy metabolism, redox homeostasis and oxidative stress level. Notably, these changes differed among the studied tissues (i.e., heart, liver and brain).

We demonstrated that strenuous endurance training enhanced mitochondrial biogenesis in all tested organs, as evidenced by increased levels of mitochondrial proteins (VDAC1 and CS) and the mitochondrial biogenesis marker protein (PGC1α). Given the average increase in the mitochondrial proteins after endurance training, it can be estimated that the increases in mitochondrial biogenesis were ~17%, ~30% and ~12% in the heart, liver and brain, respectively. An increase in mitochondrial biogenesis after exercise or chronic training has previously been demonstrated in rodent heart [27], liver [28] and brain [29]. Moreover, endurance exercise-induced systemic mitochondrial biogenesis has been observed in many tissues, including the heart, liver and brain in mtDNA mutant mice [30].

The changes in mitochondrial biogenesis observed in this study were accompanied by larger changes in the total (reduced plus oxidized) Q9 + Q10 pool (~36, ~45 and ~21% in the heart, liver and brain, respectively), indicating that the nonmitochondrial Q pool also increased in all tissues. The tissue changes induced by training in the oxidized Q pool (reducible by the respiratory chain) can mainly be attributed to an increase in mitochondrial biogenesis, while changes to the reduced Q (QH_2_) pool can be attributed to an increase in mitochondrial and nonmitochondrial antioxidants. In the heart, the total pool of reduced Qs (QH_2_9 + QH_2_10) increased by ~83%, while the total pool of oxidized Qs (Q9 + Q10) only increased by ~23%. Moreover, the increased total redox state of Q (QH_2_/Q) increased by ~50% (from 0.25 to 0.37), indicating an upregulation of the antioxidant form of Q in a trained heart. In the liver, the total reduced and oxidized Q pools increased after endurance training by ~47 and ~36%, respectively. Meanwhile, the total Q redox state increased slightly (from 4.50 to 4.82), indicating no significant training-induced changes in the Q redox state. In the case of the brain, the total oxidized Q pool increased by ~12%. However, quantitative changes in the total reduced Q pool were difficult to quantify since QH_2_ levels were not detected in control rats. However, it can be estimated that the Q redox state in the brain increased significantly after training. These results are consistent with earlier determinations of the ubiquinol/ubiquinone ratio after 8 weeks of treadmill running, which showed a ~40% and ~10% increase in this ratio within heart and brain tissues, respectively and no change in the liver [4]. Taken together, the response of QH_2_ as an antioxidant pool to exercise varies from tissue to tissue.

While ROS are involved in redox signaling, they can lead to oxidative damage when present in excess. Therefore, the amount of ROS should be controlled. At the tissue (tissue homogenate) level, a statistically significant increase in H_2_O_2_ release was only observed in the heart and only under OXPHOS inactivation conditions. These observations indicate that endurance training may increase overall heart ROS production, but not likely above safe levels since SOD1 levels were even lower in the hearts of trained rats. In the liver and brain, endurance training did not increase oxidant formation. In the brain, elevated levels of SOD1 may help to remove excess ROS. These observations support the idea that the heart may be much more exposed to exercise-induced oxidative stress than the brain and liver, which may be due to a much greater increase in oxygen consumption during exercise compared to the level at rest (as previously discussed). In the brain, it is especially important to maintain a normal redox state due to the enhanced sensitivity to ROS [31].

Studies with mitochondria isolated from control tissues and trained rats provided insights into changes induced in the mQ pool, mROS formation and respiratory chain levels. Here, we demonstrated for the first time that endurance training can increase the mQH_2_ antioxidant pool in the mitochondria of certain tissues. Endurance training increased the total reduced mQ pool (QH_2_9 + QH_2_10) in the heart and liver by ~70% and ~14%, respectively, while it did not significantly change this pool in the brain. These results suggest that the heart mitochondria of trained rats have a particularly high demand for this antioxidant. Liver mitochondria, which initially had the largest reduced mQ pool, increased the mQH_2_ pool to a lesser extent in response to training, while brain mitochondria did not use this molecule as an antioxidant under training conditions. Measurements of mROS in mitochondria isolated from control and training rats support these observations. Namely, endurance training did not increase the production of mROS in the mitochondria of the liver and brain. Depending on the involvement of individual dehydrogenases in the respiratory chain, it could increase this production in the mitochondria of the heart. In our study, no increase in the level of the antioxidant enzyme SOD2 was observed in the mitochondria of the heart, liver and brain of the trained rats.

Our research has shown that the mROS formation observed in the mitochondria of individual rat tissues after training may be associated with changes in the activity/amount of individual elements of the OXPHOS system and its molecular organization, as well as with the size of the oxidized pool of mQ acting as an electron-carrier in the respiratory chain. The enhanced energy requirements of the heart associated with endurance training performance (up to a ~5–6-fold increase in oxygen consumption from resting levels [24]) and a relatively small increase in mitochondrial biogenesis (~20%; this study) imply that major adaptive changes that can meet this metabolic challenge likely occur at the cardiac OXPHOS system level. In the heart mitochondria of trained rats, we observed an increase in the amount/activity of all components of the OXPHOS system (except the non-limiting CIV) as well as in the oxidized Q pool, which may indicate increased OXPHOS yield. Additionally, the observed rearrangement of supercomplexes in the respiratory chain of the mitochondria of trained rats, coupled with an increase in the oxidized mQ pool (~23%), may lead to a decrease in mROS production during the oxidation of CI substrates.

In the hepatic mitochondria of trained rats, rearrangement of the components of the respiratory chain and a concomitant increase in the pool of antioxidants (i.e., mQH_2_) can lead to the observed overall reduction in mROS formation. Namely, as a result of training, a decreased level/activity of the mQ-reducing pathways (Cl and CII) and an increased level of the mQH_2_-oxidizing CIII may lead to a decrease in the level of mQ reduction and thus the production of mROS. It has recently been shown that mROS formation is directly dependent on the level of mQ reduction (mQH_2_/mQtot) in the active pool associated with the respiratory chain [8]. Moreover, the increased level of UCP2 in the liver mitochondria of trained rats may be involved in lowering mROS formation.

In the mitochondria of the brain, endurance training did not alter the total reduced and oxidized mQ pools or increase the level of mROS formation. As a result of training, increased levels and/or activity of CII and CI + CIII supercomplexes may lead to no increase in mROS formation and possibly an increased OXPHOS yield since CV activity is slightly increased. Moreover, the increased level of UCP2 after endurance training observed in the brain mitochondria may be involved in maintaining unelevated mROS levels. It has previously been observed that voluntary exercise induces UCP2 mRNA expression and mitochondria oxygen consumption in phosphorylating and nonphosphorylating respiratory states in the hippocampus of mice [32].

Our research has shown that in the mitochondria of the heart, liver and brain, endurance training triggered a different response to the redox state of mQ. Namely, the mQH_2_/mQ ratio increased by 38% (from 0.24 to 0.33) and by 25% (from 1.19 to 1.49) in the heart and liver mitochondria, while remaining unchanged in the brain mitochondria (0.17). These results show for the first time that endurance training can affect mQ redox homeostasis in certain tissues. To date, it has been observed that after 8 weeks of endurance training, the total oxidized plus reduced mQ pool is lowered in lung mitochondria [8] and increased in skeletal muscle mitochondria [11].

Our results indicate that endurance training increased the nonmitochondrial and mitochondrial Q pools, especially the reduced Q pools, in all tissues tested. The question is whether Q10 supplementation could help meet the increased demand for Q in the heart, liver and brain of training animals. Perhaps increased endogenous Q synthesis in these tissues is sufficient. However, further research should clarify these points. There is also no convincing evidence that supplementation with Q10 as an antioxidant is effective and should be recommended to athletes [33]. Systemic functional measurements in several tissues, including isolated mitochondria, are difficult and limited to perform. Our measurements do not describe the actual level of mQ reduction under the reducing pressure of the active mitochondrial respiratory chain. Moreover, it would be interesting to study the total respiration rate under resting and phosphorylating conditions during the oxidation of various respiratory substrates in order to compare the total bioenergetic efficiency with the partial parameters presented on the individual complexes. Therefore, more research is needed to elucidate the effects of endurance training on respiratory chain activity in relation to the functioning of mQ as an electron carrier in the mitochondria of the heart, liver and brain, as well as in other tissues where it has not been studied.

Taken together, our results show that endurance training can induce various tissue and mitochondrial adaptive remodeling related to Q acting as an antioxidant and electron carrier in the respiratory chain. Thus, these results emphasize the role of the QH_2_ pool in protecting against excessive ROS levels and the role of the oxidized mQ pool (reduced by the respiratory chain) in the adaptation of the OXPHOS system and modulation of mROS production in response to endurance training. Several factors, including oxygen consumption, metabolic rate, susceptibility to oxidants, activation of antioxidant proteins and levels of other antioxidants, may influence organ-based differences in Q-related responses to endurance training.

## 4. Materials and Methods

### 4.1. Animals and Endurance Training 

The study was conducted in 16 adult, 4-month-old male Wistar rats that were randomly assigned to the training group (*n* = 8) or the control group (*n* = 8). During the experiment, the animals had free access to food and water and were kept under standard conditions of humidity and temperature on a 12 h light/dark cycle. Experimental protocols involving animals, their care, training and surgery were approved by the Local Ethics Committee on Animal Experimentation in Poznan, Poland (Permit Number: 15/2013) and were in compliance with the guidelines of the European Community Council Directive on the protection of animals used for scientific purposes.

The 8-week training was conducted five times per week on a treadmill for small rodents (Exer 3/6 M Treadmill, Columbus Instruments, Columbus, OH, USA) as previously described [11]. During the first week of training, rats were introduced to treadmill running at different speeds (20–30 m × min^−1^) during 20–30 min running sessions. At the end of the first week, the training session was extended to 40 min. In the first 2 weeks, the basal running speed was set at 30 m × min^−1^ and increased to 40 m × min^−1^ for 20 s every 10 min. After 4 weeks, the training sessions were extended to 1 h with a running speed of 30 m × min^−1^, which increased to 40 m × min^−1^ after ~10 min. The duration of the higher speed was gradually increased from 20 s in the 6th week to 40 s in the final week of training. The day after the end of the final training session, the exercise and control rats were sacrificed by decapitation. Every effort was made to minimize suffering. 

### 4.2. Tissue Preparation and Mitochondria Isolation

All steps of tissue preparation and mitochondria isolation were performed at 4 °C as previously described [19], with some modifications. To isolate mitochondria from rat heart, liver and brain (cortex), tissues were harvested and placed in isolation medium A (pH 7.2) containing 50 mM Tris-HCl, 100 mM sucrose and 0.5 mM ethylenediaminetetraacetic acid (EDTA) and washed several times. Blood cells were removed by decantation. Tissues were cleaned of connective tissue and large vessels, cut into small pieces on ice and then filtered. Tissues were homogenized in isolation medium B containing 50 mM Tris-HCl (pH 7.2), 100 mM sucrose, 1 mM KH_2_PO_4_, 100 mM KCl, 0.5 mM EDTA and 0.1 mM ethylene glycol-bis(β-aminoethyl ether)-N,N,N′,N′-tetraacetic acid (EGTA) using a Teflon or glass pestle at different times and intensity. The filtered homogenates were centrifuged once or twice at 900× *g* for 10 min. Some of the filtered homogenate supernatants were used for measurements (H_2_O_2_ release, the Q content and reduction level and protein immunoblotting). The remainder of the homogenates was supplemented with isolation medium B with 0.2% bovine serum albumin (BSA) and centrifuged at 17,800× *g* for 10 min. Mitochondrial pellets were suspended in isolation medium B without BSA and centrifuged at 900× *g* for 8–10 min. The supernatants were filtered and again centrifuged at 17,800× *g* for 10 min. The heart and liver mitochondria were washed and centrifuged again. The final mitochondrial pellets were then resuspended in a small volume of medium C containing 10 mM Tris-HCl (pH 7.2), 75 mM sucrose and 225 mM mannitol. 

The Bredford method was used to determine the protein concentration in homogenates and mitochondria. All functional measurements were performed at 35 °C. Measurements of H_2_O_2_ release and Q content were normalized to GAPDH expression levels (homogenates) and cytochrome *c* oxidase (COX) activity or expression levels (isolated mitochondria).

### 4.3. H_2_O_2_ Release

The Amplex Red assay was used to measure the rate of H_2_O_2_ release as previously described [19]. Measurements were performed with horseradish peroxidase (HRP, 0.14 U × mL^−1^), Amplex Red (5 µM) and exogenous superoxide dismutase (SOD, 5U × mL^−1^) to convert superoxide anion to H_2_O_2_. As inhibitor of HRP-independent conversion of Amplex Red to resorufin, 100 µM PMSF was added to the experimental medium immediately prior to the measurement [34]. The fluorescence change was followed for 40 min at 545 nm/590 nm using a Tecan multimode reader (Infinite M200 PRO) with 24-well plates. Homogenates (50 or 100 µg of protein) were incubated in 0.5 mL of standard assay medium (75 mM sucrose, 225 mM mannitol, 5 mM KH_2_PO_4_, 10 mM KCl, 0.5 mM EDTA, 10 mM Tris/HCl (pH 7.2) and 0.2% BSA) with a mixture of 5 mM succinate, 5 mM malate and 5 mM glutamate under nonphosphorylating or phosphorylating conditions (in the presence of 0.5 mM ADP). Mitochondria (0.4 mg of protein) were incubated in 0.5 mL of the standard incubation medium with 5 mM succinate, 5 mM malate and 5 mM glutamate or a mixture of these three substrates in the absence (nonphosphorylating state 4 conditions) or presence of 0.5 mM ADP (phosphorylating state 3 conditions). H_2_O_2_ release of nonphosphorylating respiration was determined after ADP depletion or in the absence of exogenous ADP. 

### 4.4. Q Content in Tissues and Mitochondria

Extraction and HPLC detection techniques were used to determine the concentration of oxidized (at 290 nm) and reduced (at 275 nm) forms of Q9 and Q10 in rat tissues and mitochondria as previously described [19]. The oxidized (Q9 and Q10) and reduced (Q9H_2_ and Q10H_2_) Q pools were determined in rat tissues and mitochondria under fully oxidizing conditions, i.e., in the absence of respiratory Q-reducing substrates. Before Q extraction, homogenates (3 mg) and mitochondria (0.8 mg) were incubated with gentle agitation for 30 min in 3 mL of the standard assay medium (at 35 °C) to obtain fully oxidizing conditions. The total oxidized and reduced Q9 + Q10 pool (Q + QH_2_) and the total Q redox state (QH_2_/Q) were then calculated.

### 4.5. Cytochrome c Oxidase Activity

The COX activity was assessed polarographically in 0.5 mL of the standard assay medium with 0.4 mg of mitochondrial protein with successively added 10 μM antimycin A, 7 mM ascorbate, 0.05% cytochrome *c* and up to 1.5 mM of N, N, N′N′-tetramethyl-*p*-phenylenediamine (TMPD). 

### 4.6. Protein Level Immunodetection

Homogenate and mitochondrial proteins were separated on 6–12% SDS-PAGE gels with the PageRuler PrestainedTM Protein Ladder (Thermo Fisher Scientific) as a marker of molecular weight as previously described [8]. We used primary Abcam antibodies raised against: CS (46 kDa) (ab96600), VDAC1 (35 kDa) (ab14734), PGC1α (92 kDa) (ab54481), SOD1 (18 kDa) (ab13498), GAPDH (37 kDa) (ab9485), UCP2 (33 kDa) (ab97931), UCP3 (34 kDa) (ab3477) and CoQ10B (46 kDa) (ab41997). Abcam total rodent OXPHOS antibody cocktails (ab110413) were used, which contained antibodies against subunits of CI (20 kDa subunit NDUFB8), CII (30 kDa subunit SDHB), CIII (subunit Core 2, 48 kDa), CIV (COXI, 40 kDa) and ATP synthase (CV) (subunit α, 57 kDa). We also used primary antibodies from other manufacturers raised against: SOD2 (25 kDa) (ADI-SOD, Enzo Life Sciences), UCP4 (25 kDa) (PA5-100668, Invitrogen) and UCP5 (33 kDa) (PA5-89394, Invitrogen). As loading controls, the expression levels of GAPDH (homogenates) and COX or CS (mitochondria) were used. Protein levels were digitally quantified using ImageJ software.

### 4.7. BN-PAGE and in-Gel Activity Assays

BN-PAGE separation of mitochondrial proteins (50–200 µg) and in-gel activity assays of complexes CI, CII, CIV and CV were performed as previously described [8,35,36]. To determine the OXPHOS complexes with immunoblotting, the BN-PAGE-separated proteins were transferred onto nitrocellulose membranes and immunodetected with anti-UQCRC2 antibody (against CIII, ab14745, Abcam) or the total OXPHOS rodent antibody cocktail (ab110413, Abcam).

### 4.8. Statistical Analysis

The means ± SD obtained from at least 3–6 independent homogenate preparations or mitochondrial isolations are presented. Each determination was performed at least in duplicate. Significant differences were determined via unpaired *t* tests or ANOVAs (followed by Tukey’s post hoc comparisons for *p* < 0.05 from an ANOVA). Differences were considered to be statistically significant if *p* < 0.05 (* or ^#^), *p* < 0.01 (** or ^##^), or *p* < 0.001 (*** or ^###^). Statistical analysis was performed with Origin v 8.5.1 software (OriginLab Corporation, Northampton, MA, USA). 

## Data Availability

The data presented in this study are openly available in Mendeley Data, V1, doi:10.17632/gm4bj3mnrw.1.
